# MicroRNA-7 targets Nod-like receptor protein 3 inflammasome to modulate neuroinflammation in the pathogenesis of Parkinson’s disease

**DOI:** 10.1186/s13024-016-0094-3

**Published:** 2016-04-16

**Authors:** Yan Zhou, Ming Lu, Ren-Hong Du, Chen Qiao, Chun-Yi Jiang, Ke-Zhong Zhang, Jian-Hua Ding, Gang Hu

**Affiliations:** Jiangsu Key Laboratory of Neurodegeneration, Department of Pharmacology, Nanjing Medical University, 140 Hanzhong Road, Nanjing, Jiangsu 210029 China; Nanjing Medical University Hospital, 300 Guangzhou Road, Nanjing, Jiangsu 210029 China; Biomedical Functional Materials Collaborative Innovation Center, College of Chemistry and Materials Science, Nanjing Normal University, Nanjing, Jiangsu 210023 China; Department of Pharmacology, Nanjing University of Chinese Medicine, 138 Xianlin Avenue, Nanjing, Jiangsu 210023 China

**Keywords:** microRNA-7, NLRP3 inflammasome, α-Synuclein, Neuroinflammation, Parkinson’s disease

## Abstract

**Background:**

α-Synuclein (α-Syn), a pathological hallmark of Parkinson’s disease (PD), has been recognized to induce the production of interleukin-1β in a process that depends, at least in vitro, on nod-like receptor protein 3 (NLRP3) inflammasome in monocytes. However, the role of NLRP3 inflammasome activation in the onset of PD has not yet been fully established.

**Results:**

In this study, we showed that NLRP3 inflammasomes were activated in the serum of PD patients and the midbrain of PD model mice. We further clarified that α-syn activated the NLRP3 inflammasome through microglial endocytosis and subsequent lysosomal cathepsin B release. Deficiency of caspase-1, an important component of NLRP3 inflammasome, significantly inhibited α-syn-induced microglia activation and interleukin-1β production, which in turn alleviated the reduction of mesencephalic dopaminergic neurons treated by microglia medium. Specifically, we demonstrated for the first time that *Nlrp3* is a target gene of microRNA-7 (miR-7). Transfection of miR-7 inhibited microglial NLRP3 inflammasome activation whereas anti-miR-7 aggravated inflammasome activation in vitro. Notably, stereotactical injection of miR-7 mimics into mouse striatum attenuated dopaminergic neuron degeneration accompanied by the amelioration of microglial activation in MPTP-induced PD model mice.

**Conclusions:**

Our study provides a direct link between miR-7 and NLRP3 inflammasome-mediated neuroinflammation in the pathogenesis of PD. These findings will give us an insight into the potential of miR-7 and NLRP3 inflammasome in terms of opening up novel therapeutic avenues for PD.

**Electronic supplementary material:**

The online version of this article (doi:10.1186/s13024-016-0094-3) contains supplementary material, which is available to authorized users.

## Background

Parkinson’s disease (PD), the second most common neurodegenerative disorder after Alzheimer’s disease, is characterized by the progressive loss of dopaminergic (DA) neurons in substantia nigra compacta (SNc), accumulation of α-synuclein (α-Syn) in Lewy bodies and neurites, and excessive neuroinflammation [1, 2]. α-Syn, a known danger-associated protein, has been shown to activate microglia and subsequently induce neuroinflammation, which plays a crucial role in the pathogenesis of PD [3–5]. Although fibrillar α-Syn could induce the synthesis of interleukin-1β (IL-1β) through interaction with Toll-like receptor 2 (TLR2) and activation of inflammasome in monocytes in vitro [6], the role of α-Syn-induced inflammasome activation in the onset of PD has not yet been fully established [7]. So far, there is no direct evidence that nod-like receptor protein 3 (NLRP3) inflammasome is involved in α-Syn-induced microglial activation and neuroinflammation in PD pathogenesis.

Microglia are the chief innate immune cells within the CNS, but they are complemented by CNS-derived macrophages that are located in the meninges, choroid plexus and perivascular space [8]. These cells constantly survey the proximal environment through the pattern-recognition receptors that they express, including Toll-like receptors and NOD-like receptors (NLRs) [9]. Activated microglia produce a large number of inflammatory cytokines that contribute to DA neuronal degeneration. Among these inflammatory cues, IL-1β has been recognized to be essential for initiation and progress of PD [10, 11]. Enhanced expression of IL-1β has been observed in the microglia surrounding Lewy bodies in PD patients as well as in animal models [11–13]. It has been known that the protease caspase-1 is critically involved in inflammatory responses due to its pivotal role in regulating the cleavage of the inactive precursor pro-IL-1β and pro-IL-18 to matured IL-1β and IL-18 in the cytosol by a variety of stimuli [14]. The activity of caspase-1 is tightly controlled by cytosolic multiprotein complexes called ‘inflammasomes’, which are composed of the nod-like receptor protein (NLRP) family, adaptor protein ASC and proinflammatory precursor pro-caspase-1 [14]. Inflammasomes containing NLRP3 are highly expressed in microglia and essential to the process of neuroinflammation [15]. NLRP3 inflammasome activation has been detected in a number of neurodegenerative diseases, including Alzheimer’s disease and amyotrophic lateral sclerosis [7]. Endogenous ‘danger’ signals such as urate crystals, bacterial toxins or beta-amyloid aggregates, intensely activate NLRP3 inflammasomes [15–17]. On the other hand, the inflammasome might be involved in the pathogenesis of PD and be developed to a potential target for PD therapy [18]. However, no other studies have addressed the possible involvement of the inflammasome in PD, except for a report describing the protective effect of P2X7 receptor blockers in a rat model of this condition [18, 19].

Emerging evidence demonstrates that post-transcriptional regulation by microRNAs (miRs) is critical in PD pathogenesis [20]. Specifically, miR-7 is evolutionarily conserved among vertebrates, including mouse and human. It has been known that miR-7 directly regulates α-Syn expression in DA neurons via post-transcriptional regulation and is associated with the pathophysiology of PD [20]. Therefore, it is also attractive to clarify whether miR-7 could directly modulate microglial NLRP3 inflammasome besides targeting α-Syn. In this study, we prepared 1,2,3,6-methyl-phenyl-tetrahydropyridine/probenecid (MPTP/p) PD model and detected NLRP3 inflammasome activation in the serum of PD patients so as to explore the role of NLRP3 inflammasome in PD pathogenesis. Furthermore, α-Syn overexpression (A53T mutation, A53T^tg/tg^) and A53T^tg/tg^;Caspase-1^-/-^ double transgenic mice were used to clarify the effects of α-Syn and miR-7 on the activation of NLRP3 inflammasome and neuroinflammation. Our study provides a direct link between NLRP3 inflammasome activation and PD pathogenesis. We further unravel that miR-7 targets *Nlrp3* expression besides α-Syn and modulates NLRP3 inflammasome-mediated inflammation.

## Results

### α-Syn triggers the NLRP3 inflammasome activation in BV2 cells

**Fig. 1:**
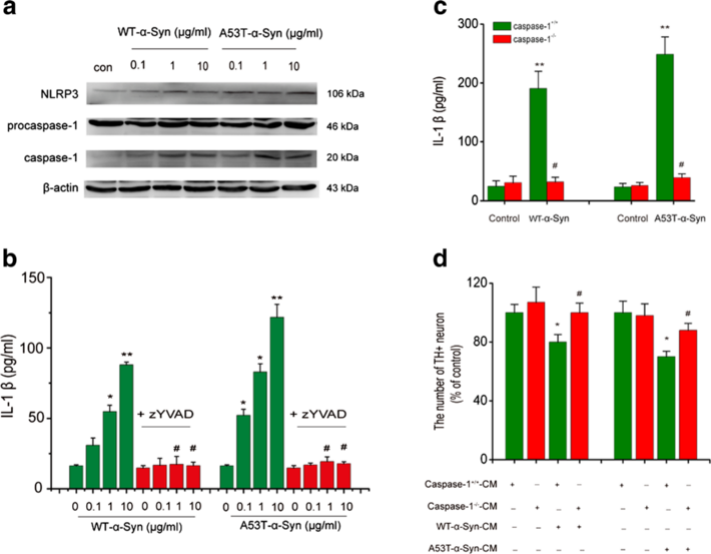
α-Synuclein activates the NLRP3 inflammasome in BV2 cells. **a** Wide-type and A53T mutants showing α-Syn induces the up-regulation of NLRP3 and Caspase-1 in BV2 cells. Data are presented as the mean ± S.E.M from four independent experiments. **b** Caspase-1-specific inhibitor (z-YVAD, 10 μM) suppresses IL-1β formation induced by both A53T mutant and wide-type α-Syn. LPS + ATP were used for a positive control drug. Data are presented as the mean ± S.E.M from four independent experiments. **c** Caspase-1 knockout abolishes WT or A53T α-Syn (10 μg/ml) induced elevation of IL-1β release in primary cultured microglia. **d** Midbrain TH neurons showed an attenuated impairment and a resistance to condition medium (CM) that was obtained from Caspase^-/-^ microglia treated with WT or A53T α-Syn. Data are presented as the mean ± S.E.M from four independent experiments. * *p* < 0.05, ** *p* < 0.01 vs. control group, # *p* < 0.05, ## *p* < 0.01 vs. α-Syn treatment group

We initially determined the roles of NLRP3 inflammasome in α-Syn-induced microglial activation and DA neuron injury in an immortalized murine microglial cell line, BV-2. This line was chosen as it has been defined that BV-2 cell line is an ideal alternative model system for primary microglia cultures [21]. After treatment with α-Syn, either wild type (WT, 1, 10 μg/ml) or A53T mutant α-Syn (0.1, 1, 10 μg/ml) increased p65 nuclear translocation and pro-IL-1β generation in BV2 cells (Additional file 1: Figure S1a-S1c). Moreover, the protein expressions of NLRP3 and caspase-1 were significantly increased whereas the protein level of pro-caspase-1 was not altered (Fig. 1a and Additional file 1: Figure S1d-S1f). Given that aggregated α-synuclein, rather than monomeric α-synuclein, has been well accepted to activate inflammasome in macrophage, we also determined the effect of fibrillary α-synuclein on inflammasome activation in BV2 cells. We prepared aggregated α-synuclein as described by Codolo et al. [6] and found that fibrillary α-synuclein also activated NLRP3 inflammasome and resulted in IL-1β secretion (Additional file 2: Figure S2a-S2e). This result indicates that both aggregated and monomeric α-synuclein can activate NLRP3 inflammasome in BV2 cells. Furthermore, previous studies also proposed that monomeric α-synuclein could induce inflammatory response in BV2 cells and microglial phagocytosis is enhanced by monomeric a-synuclein, not aggregated a-synuclein. We thus selected monomeric α-synuclein in the following experiments. Notably, pharmacological blockage of caspase-1 with zYVAD (10 μM) abolished α-Syn-induced IL-1β elevation (Fig. 1b), suggesting that α-Syn stimulated IL-1β generation in inflammasome-dependent manner.

Subsequently, caspase-1 knockout mice were used to investigate the roles of inflammasome activation in α-Syn-induced microglial inflammation and DA neuron degenerative damage. We treated primary microglia isolated from wild type and caspase-1^-/-^ mice with WT or A53T α-Syn (10 μg/ml) for 24 h and measured IL-1β levels in the supernatants. As a result, both WT and A53T α-Syn induced a marked (roughly 10-fold) elevation of IL-1β in cultured midbrain microglia prepared from WT, but not from caspase-1^-/-^ mice (Fig. 1c). Furthermore, we examined toxic effects of the conditioned medium from both genotypic microglia on primary culture of midbrain TH positive neurons using cell counting. The results showed that only the conditioned medium from WT microglia treated with α-Syn, but not caspase-1^-/-^ microglia cultures significantly reduced midbrain TH positive neuronal numbers (Fig. 1d). These findings suggest that inflammasome is at least, in part a contributing factor causing the loss of TH neurons. Together, these results show that α-Syn can activate NLRP3 inflammasomes, which in turn promotes IL-1β production and leads to DA neuron injury.

### NLRP3 inflammasome activation by α-Syn is endocytosis-dependent

**Fig. 2:**
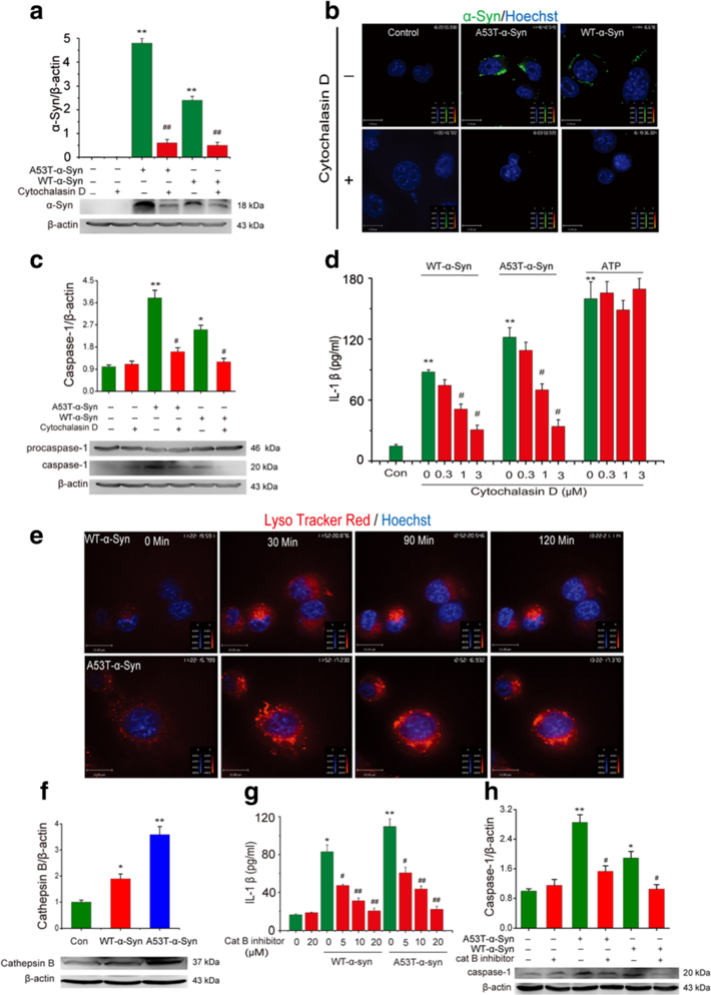
α-Syn activates NLRP3 inflammasome via endocytosis and lysosomal impairment. **a** Immunoblot analysis of α-Syn in BV2 cells stimulated with A53T mutant or wide-type α-Syn, cytochalasin D (3 μM) inhibits endocytosis of α-Syn into BV2 cells. Data are presented as the mean ± S.E.M from four independent experiments. **b** Confocal microscopy of immortalized microglia BV2 cells incubated for 4 h with FITC-labeled α-Syn (10 μM) and then processed for immunocytochemistry, cell nuclei were visualized with hoechst. Data are presented as the mean ± S.E.M from three independent experiments. **c** Immunoblot analysis of caspase-1 in BV2 cells stimulated with A53T mutant or wide-type α-Syn, cytochalasin D (3 μM) inhibits activation of caspase-1 in a concentration-dependent manner in BV2 cells. Data are presented as the mean ± S.E.M from three independent experiments. **d** ELISA of the release of IL-1β into supernatants of BV2 cells treated with cytochalasin D during stimulation with A53T mutant or wide-type α-Syn or ATP. Data are presented as the mean ± S.E.M from four independent experiments. **e** α-Syn-containing lysosomes adopt a swollen morphology and underwent structural damage once α-Syn was phagocytosed by BV2 cells. Data are presented as the mean ± S.E.M from three independent experiments. **f** WT or A53T α-Syn in turn triggers the release of lysosomal protease cathepsin B into the cytoplasm. **g**-**h** Cathepsin B inhibitor significantly suppresses α-Syn evoked increase of caspase-1 expression and IL-1β production. Data are presented as the mean ± S.E.M from four independent experiments. * *p* < 0.05, ** *p* < 0.01 vs. control group, # *p* < 0.05, ## *p* < 0.01 vs. α-Syn treatment group

We next studied the underlying mechanisms by which α-Syn entered into BV2 cells during α-Syn exposure. As shown in Fig. 2a, incubation of BV2 cells with WT or A53T α-Syn for 24 h resulted in a visible increase of α-Syn levels in the cytoplasm, which was detected by Western blotting. Pharmacological blockage of endocytosis with cytochalasin D (3 μM), an inhibitor of endocytosis, abolished this upregulation of α-Syn in the cytoplasm, suggesting that α-Syn may enter cell through endocytosis. This notion was further supported by confocal microscopy imaging, where FITC-labeled α-Syn (10 μM) accumulated in the cytoplasm after 4 h exposure (Fig. 2b). Since ATP activates NLRP3 inflammasome via P2X7 receptors in the cytomembrane and is endocytosis-independent [22], we compared the effects of cytochalasin D on α-Syn- and ATP-induced elevation of IL-1β. We found that cytochalasin D eliminated both α-Syn-induced caspase-1 activation in BV2 cells (Fig. 2c) and IL-1β elevation in culture medium (Fig. 2d), but failed to suppress ATP-induced IL-1β production. These results indicate that α-Syn enters into BV2 cells in an endocytosis- dependent manner and subsequently triggers NLRP3 inflammasome activation.

### α-Syn activates NLRP3 inflammasome via inducing lysosomal swelling and increasing cathepsin B release

To investigate how α-Syn activates NLRP3 inflammasomes through the endocytosis, we monitored the dynamic alteration of lysosomes as the functional impairment of lysosomes consistently activates NLRP3 inflammasomes [23]. Incubation of BV2 cells with α-Syn for 2 h triggered lysosomal swelling (Fig. 2e) and increased cathepsin B expression in the cytoplasm (Fig. 2f). As a specific inhibitor of cathepsin B, Z-FA-FMK (20 μM) could eliminate both α-Syn-induced activation of caspase-1 in BV2 cells (Fig. 2h) as well as the elevation of IL-1β release in culture medium (Fig. 2g). These results suggest that lysosomal damage is involved in the α-Syn-induced activation of NLRP3 inflammasomes. Given that lysosomal function is tightly associated with autophagy [24], we further examined the effects of α-Syn on autophagy and subsequent intracellular ROS accumulation. As expected, α-Syn could inhibit AMPK phosphorylation (Additional file 3: Figure S3a) and suppressed the ratio of LC3-II to LC3- I (Additional file 3: Figure S3b), and these inhibitory effects were reversed by 5-amino-1-β-D-ribofuranosyl-imidazole-4-carboxamide (AICAR, 100 μM), a selective AMPK activator. Meanwhile, α-Syn drastically increased ROS accumulation in BV2 cells and this effect was attenuated by either cytochalasin D (3 μM) or AICAR (Additional file 3: Figure S3c & S3d). It was also found that AICAR could abolish the increase of cathepsin B induced by WT- or A53T-α-synuclein. This result indicates an important role of AMPK in α-synuclein-induced lysosomal damage (Additional file 3: Figure S3e). Moreover, AICAR could also abolish α-Syn-induced activation of caspase-1 (Additional file 3: Figure S3f) and the increase of IL-1β production (Additional file 3: Figure S3g). These results demonstrate that the microglial endocytosis of α-Syn, the lysosome damage and the AMPK phosphorylation-dependent ROS accumulation underlie the α-Syn-induced activation of NLRP3 inflammasomes.

### Inflammasome is activated in serum of PD patients and in the midbrain of α-Syn-overexpressed mice

**Fig. 3:**
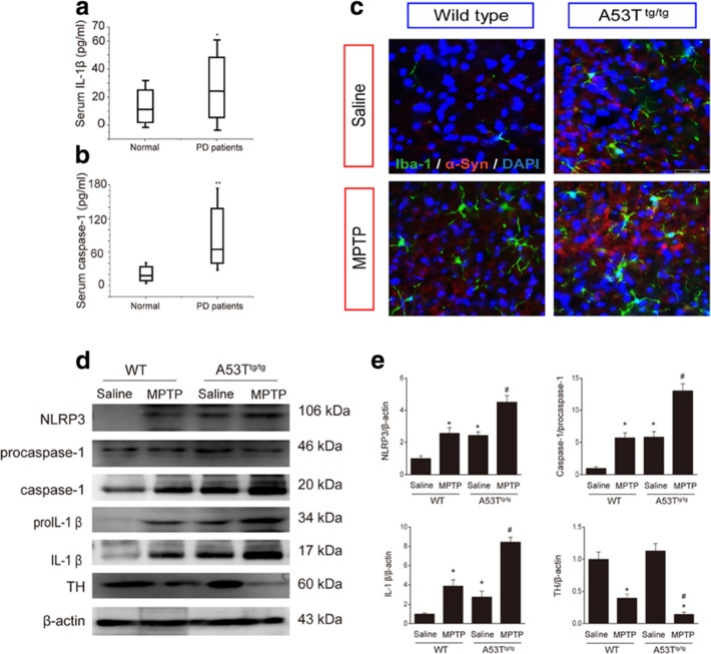
Inflammasome is activated in serum of PD patients and in the midbrain of α-Syn-overexpressed mice. **a** Serum levels of IL-1β and **b** caspase-1 activity are upregulated in PD patients. * *p* < 0.05, ** *p* < 0.01 vs. normal group. Data are presented as the mean ± S.E.M, *n* = 12. **c** Immunofluorescence exhibits enhanced α-Syn accumulation and aggravated Iba-1^+^ microglial immunofluorescence intensity in the SNc of A53T transgenic mice. Green: Iba-1, red: α-Syn, blue: DAPI, *n* = 5–6. **d**-**e** A53T mutant mice exhibit aggravated activation of NLRP3 inflammasome and the lower levels of TH expression in the midbrain in the presence or absence of MPTP injections, *n* = 4. * *p* < 0.05 vs. wild type group, # *p* < 0.05 vs. A53T^tg/tg^ mice

Next, we explored the role of NLRP3 inflammasome activation in the pathogenesis of PD. The serum samples of twelve PD patients (age 63 ~ 78-year old, evenly divided between men and women) without drug therapy were collected to detect inflammasome activation. As expected, these PD patients exhibited a remarkable elevation of serum IL-1β levels accompanied by an enhancement of caspase-1 activity compared to age-matched healthy controls (Fig. 3a & b). These data provide a direct evidence that inflammasome activation involves the pathogenesis of PD. Given that α-Syn activated NLRP3 inflammasome-mediated microglial inflammation in in-vitro, we further investigated the effect of α-Syn on NLRP3 inflammasome activation in in-vivo. Consistently, A53T transgenic mice displayed a dramatic aggravation of microglial activation accompanied by the α-Syn accumulation in the presence or absence of MPTP treatment as determined by immunofluoresence staining (Fig. 3c). Furthermore, it was found the expression of NLRP3, caspase-1 and the level of matured IL-1β in midbrain were significantly increased in A53T transgenic mice in basal situation. At the end of MPTP administration, A53T^tg/tg^ mice exhibited the aggravated activation of NLRP3 inflammasome and IL-1β production, as well as the lower level of TH expression, compared with those in WT mice (Fig. 3d-e). Thus, it is reasonable that activation of the NLRP3 inflammasomes may play an important role in PD pathogenic process.

### Caspase-1 deficiency attenuates α-Syn-induced microglial activation in mouse SNc

**Fig. 4:**
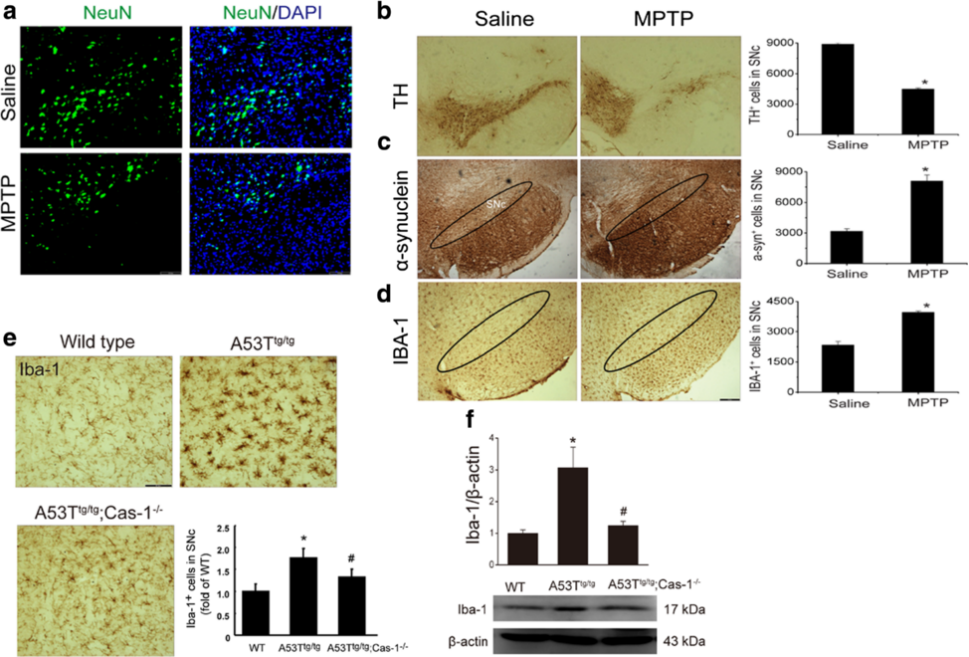
Caspase-1 deficiency attenuates α-Syn-induced microglial activation in mouse SNc. **a** NeuN immunostaining exhibits a decrease of total neurons in the SNc of MPTP/p mice. Stereological counts reveal that MPTP treatment for 5-week results in a loss of TH^+^ DA neurons in SNc (**b**) and a dramatic increase of α-Syn-ir cells accumulation (**c**). **d** Concomitantly, MPTP induces the overactivation of microglia which was characterized by the increased Iba-1-ir cells in SNc. Scale bar: 150 μm, data are presented as the mean ± S.E.M, *n* = 5. **e** IBA-1^+^ microglial activation and **f** IBA-1 expressions in WT, A53T alone transgenic mice and A53T^tg/tg^;Caspase-1^-/-^ double transgenic mice. Scale bar: 40 μm, data are presented as the mean ± S.E.M, *n* = 4–6. * *p* < 0.05 vs. saline group

We next prepared MPTP/p mouse model and showed a decrease of total neurons (NeuN^+^ cells) in the SNc in our established MPTP/p model mice (Fig. 4a). Especially, tyrosine hydroxylase positive (TH^+^) DA neuronal degeneration (Fig. 4b) was accompanied by a visible accumulation of α-Syn (Fig. 4c) and microglial activation (Iba-1 immunostaining, Fig. 4d). These results indicated a possible link between α-Syn and microglial activation. Therefore, A53T^tg/tg^ mice were used to determine the effect of α-Syn overexpression on microglial activation, and A53T^tg/tg^;Caspase-1^-/-^ double transgenic mice were used to further clarify the roles of NLRP3 inflammasomes in α-Syn-evoked neuroinflammation. In agreement with the findings in MPTP/p model, A53T^tg/tg^ mice showed an increased number of IBA^+^ microglia and a swelling of microglial morphology (Fig. 4e). Notably, A53T^tg/tg^;Caspase-1^-/-^ mice exhibited a marked amelioration of microglial activation, evidenced by the decrease in IBA-1^+^ cell number (Fig. 4e) and downregulation of IBA-1 expression (Fig. 4f) in SNc compared with A53T^tg/tg^ mice. These results demonstrate that α-Syn induces microglia-mediated neuroinflammation via activating NLRP3 inflammasome.

### *Nlrp3* is a target gene of miR-7

**Fig. 5:**
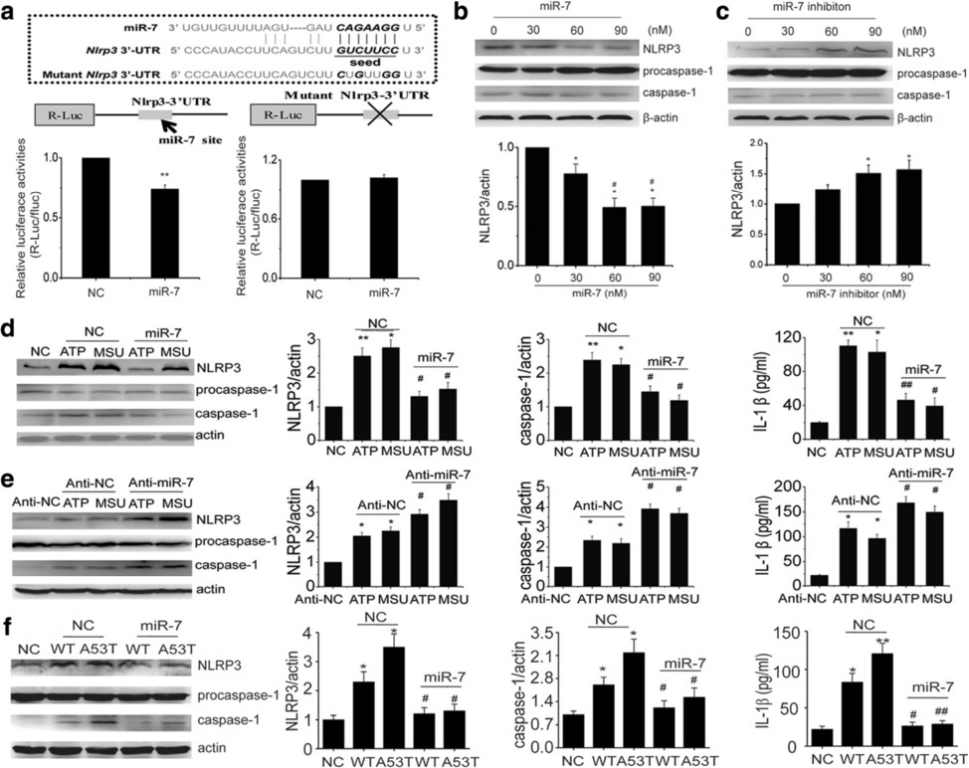
Nlrp3 is a target gene of microRNA-7. **a** Luciferase reporter assays confirm that Nlrp3 is a direct target gene of miR-7. Data are presented as the mean ± S.E.M from three independent experiments. **b** Transfection of miR-7 into BV2 cells significantly reduces NLRP3 expression but fails to affect procaspase-1 and caspase-1 expressions. **c** Anti-miR-7 upregulates NLRP3 expression but has no effect on caspase-1 maturation. Data are presented as the mean ± S.E.M from four independent experiments. **d** Transfection of miR-7 into BV2 cells markedly suppresses ATP or MSU triggerd upregulation of NLRP3 and caspase-1 as well as inhibits IL-1β production. **e** In contrast, miR-7 inhibitor exacerbates inflammasome activation characterized by aggravated NLRP3, caspase-1 expression and IL-1β release upon the stimulus of ATP or MSU. **f** Transfection of miR-7 suppresses WT or A53T α-Syn-induced upregulation of NLRP3 and caspase-1 as well as reduces IL-1β production. Data are presented as the mean ± S.E.M from four independent experiments. * *p* < 0.05, ** *p* < 0.01 vs. control or NC group, # *p* < 0.05, ## *p* < 0.01 vs. 30 nM of miR-7, anti-miR-7 treatment or corresponding NC plus ATP, MSU or α-Syn group

Emerging evidence demonstrates that post-transcriptional regulation by microRNAs is critical in PD pathogenesis [20]. Specifically, miR-7 is evolutionarily conserved among vertebrates, including mouse and human. It has been known that miR-7 targets α-Syn in DA neurons and is associated with the pathophysiology of PD [20]. In our study, α-Syn accumulation was detected in the midbrain of MPTP mouse compared with that in saline-treated mouse (Additional file 4: Figure S4a). Excitingly, it was predicted that NLRP3 might be a target gene of miR7 by employing the widely used program, miRanda [25], suggesting that miR-7 might play an additional role besides regulating α-Syn. Thus, it is interesting in test whether microglia expresses miR-7 and altered miR-7 can modulate microglial NLRP3 inflammasome. As expected, we found that miR-7 was expressed in both primary microglia and BV2 cells (data not shown). To further verify our prediction, we inserted the 3′-untranslated region (3′-UTR) sequence of NLRP3 mRNA containing a prominent miR-7 seed sequence into a dual luciferase reporter construct, which allowed us to assess NLRP3 protein translation based on luciferase activities. We discovered that miR-7 could suppress the expression of Renilla luciferase (R-Luc) through the NLRP3 3′-UTR. When miR-7 seed sequence within the R-Luc-NLRP3-3′-UTR reporter was mutated, the miR-7-mediated suppression of R-Luc reporter activity was abolished (Fig. 5a). Next, we determined the effect of altered miR-7 or anti-miR-7 on endogenous NLRP3 expression in BV2 cells. The results showed that the transfection of miR-7 significantly reduced NLRP3 protein levels (Fig. 5b), whereas anti-miR-7 transfection up-regulated NLRP3 expression (Fig. 5c). Neither miR-7 nor anti-miR-7 transfection altered the protein levels of caspase-1 and IL-1β production (Additional file 4: Figure S4b-S4f). These results demonstrate that *Nlrp3* is a target gene of miR-7.

### miR-7 inhibits NLRP3 inflammasome activation upon the stimulus in vitro

To uncover whether miR-7 controls the activity of NLRP3 inflammasomes, we further assessed the effects of miR-7 or anti-miR-7 on ATP- or MSU-induced activation of NLRP3 inflammasomes in BV2 cells. Transfection of miR-7 mimic effectively suppressed caspase-1 activation and subsequent IL-1β production induced by ATP and MSU (Fig. 5d). Whereas, anti-miR-7 transfection exacerbated MSU or ATP-induced activation of NLRP3 inflammasomes (Fig. 5e). Given that α-synuclein-triggered neuroinflammation plays a crucial role in the pathogenesis of PD, we also detected the effect of miR-7 on NLRP3 inflammasome activation induced by α-synuclein. In agreement with the results of ATP and MSU, miR-7 also can inhibit α-synuclein-induced NLRP3 upregulation and inflammasome activation (Fig. 5f). These results indicate that miR-7 negatively regulates NLRP3 inflammasome activation induced by a variety of stimuli via different pathways.

### miR-7 mimics suppress NLRP3 inflammasome activation and protects DA neurons against degeneration in PD model mice

**Fig. 6:**
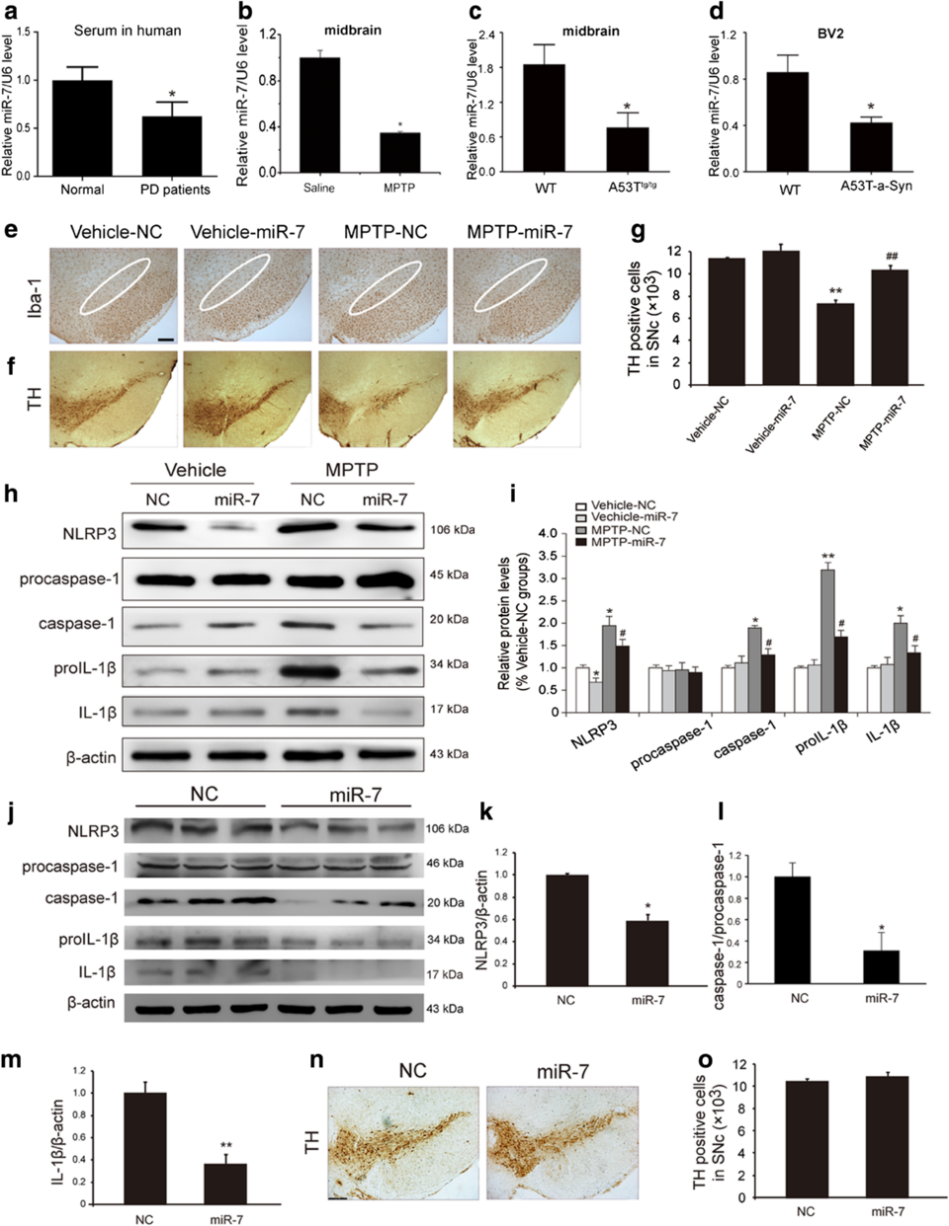
miR-7 inhibits NLRP3 inflammasome activation and attenuates DA neuronal loss in vivo. Relative miR-7 levels in the serum samples of PD patients (**a**, *n* = 12), the midbrain of MPTP-treated mice (**b**) and A53T^tg/tg^ mice (**c**) as well as α-Syn-treated BV2 cells (**d**). Data are presented as the mean ± S.E.M from four independent experiments. **e** miR-7 dramatically decreases the activation of IBA-1^+^ microglia in WT mice treated with MPTP. Scale bar: 150 μm (**f**-**g**) miR-7 mimics remarkedly increase the number of TH^+^ neurons in the SNc of WT mice treated with MPTP. Data are presented as the mean ± S.E.M, *n* = 6. **h**-**i** Injection of miR-7 mimics obviously inhibited NLRP3 inflammasome activation in MPTP-treated mice. **j**-**m** Stereotactic injection of miR-7 mimics into the striatum of A53T mice significantly downregulates NLRP3 expression and inhibits production of caspase-1 and IL-1β in vivo. **n**-**o** immunostaining showed miR-7 mimics had no significant effect on TH^+^ cell number in A53T^tg/tg^ mice at basal state. Data are presented as the mean ± S.E.M, *n* = 4. * *p* < 0.05, ** *p* < 0.01 vs. NC or vehicle group, # *p* < 0.05, ## *p* < 0.01 vs MPTP-NC group

We further investigated the role of miR-7 in the pathogenesis of PD. Firstly, we detected serum samples and found that the serum samples of PD patients showed a 30 % decrease in miR-7 levels compared with healthy controls (Fig. 6a). Consistently, in the MPTP/p-treated mice and A53T^tg/tg^ mice, miR-7 levels were reduced by about 60 % and 55 % respectively, in the midbrain detected by real time PCR (Fig. 6b & c). Moreover, α-synuclein treatment also induced an obvious reduction of miR-7 level in BV2 cells (Fig. 6d). These findings suggest miR-7 may exert a crucial role in the pathogenesis of PD. Given that miR-7 could inhibit NLRP3 inflammasome activation in vitro, we thus stereotactically injected miR-7 mimics into wild type mice that treated with subacute MPTP challenge to evaluate the protective effect of miR-7 on DA neurons. Most notably, injection of miR-7 mimics dramatically inhibited IBA-1^+^ microglial activation (Fig. 6e) and rescued the loss of TH^+^ neuron number in the SNc of MPTP-treated mice (Fig. 6f - g). miR-7 mimics had no effect on the numbers of IBA-1^+^ and TH^+^ cells in mice without MPTP administration. Meanwhile, miR-7 mimics significantly inhibited NLRP3 inflammasome activation and IL-1β production in MPTP-treated mice (Fig. 6h & i). Finally, we injected miR-7 mimics into the striatum of A53T^tg/tg^ mice to assess its effect on NLRP3 inflammasome in in vivo. Western blotting analysis showed that miR-7 mimics significantly down-regulated midbrain NLRP3 expression accompanied by the inhibition of caspase-1 activation and reduction of IL-1β production (Fig. 6j-m), while immunostaining showed miR-7 mimics had no significant effect on TH^+^ cell number in A53T^tg/tg^ mice at basal state (Fig. 6n & o). These data further demonstrate that miR-7 protects DA neurons against PD-like degeneration via suppressing NLRP3 inflammasome-mediated neuroinflammation.

## Discussion

**Fig. 7:**
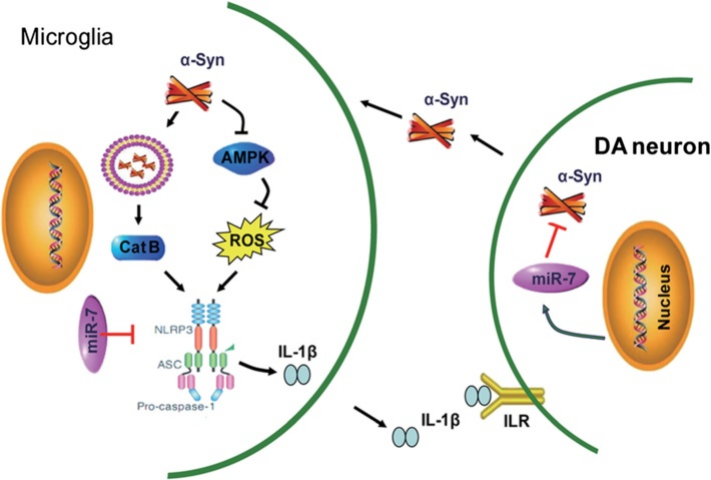
Schematic illustration demonstrates that microglial NLRP3 inflammasome mediates α-Syn-induced neuroinflammation and DA neuron degeneration in MPTP/p PD mouse model. miR-7 targets *Nlrp3* expression and modulates neuroinflammation in the pathogenesis of PD

Although α-Syn has been known to induce neuroinflammation and to correlate with PD process, the exact events underlying α-Syn-mediated microglia activation are not clarified clearly. Herein, we demonstrate for the first time that NLRP3 inflammasome activation leads to microglia-mediated neuroinflammation and DA neuronal degeneration, palying a critical role in PD pathogenesis. We further illustrate that α-Syn activates NLRP3 inflammasomes by microglial endocytosis and lysosome impairment. Moreover, in vitro and in vivo evidence shows that miR-7, a known microRNA involved in PD via regulating α-Syn, targets *Nlrp3* expression and inhibits NLRP3 inflammasome activation in PD model mice (Fig. 7). Our study provides a direct link between miR-7 and NLRP3 inflammasome-mediated neuroinflammation in the pathogenesis of PD.

IL-1β has been regarded as a critical inducer in the pathogenesis of neurodegenerative diseases, including AD and PD [26, 27]. The NLR family member NLRP3, along with the adaptor protein ASC, can activate caspase-1 via inflammasome assembly and result in the production of mature IL-1β and IL-18 [9, 27]. Thus far, a variety of particulates and crystalline substances have been identified as agonists for the NLRP3 inflammasome, such as ATP, monosodium urate crystals (MSU), silica, calcium pyrophosphate dihydrate crystals, asbestos, aluminum hydroxide, bacterial toxins and malaria hemozoin [28]. Especially, it has been demonstrated that activation of NLRP3 inflammasome by neuronal Aβ is responsible for the development of AD [15, 27]. We here find that NLRP3 inflammasome is also assembled and activated in both PD patients and PD mice, characterized by the elevated caspase-1 activity and IL-1β level in the serum of PD patients and the upregulated NLRP3 expression as well as matured IL-1β in the midbrain of PD mice. These findings imply that activation of the NLRP3 inflammasome plays a crucial role in PD pathogenesis.

The innate immune system acts at the frontline of the broader immune response through the sensing of pathogen-associated molecular patterns (PAMPs) and danger-associated molecular patterns (DAMPs) on infectious agents or disease-associated host molecules by pattern-recognition receptors [9]. In the CNS, pattern-recognition receptors are primarily expressed by microglia and astrocytes, and only cytosolic receptors are involved in the formation of inflammasomes [9]. Multiple DAMPs have been demonstrated to activate NLRP3 inflammasome and to evoke neuroinflammation [9, 28]. We thus propose that in central nervous system, α-Syn may represent a class of endogenous DAMPs to activate inflammasome like its effect on monocytes. In line with this hypothesis, our results demonstrate that α-Syn leads to NLRP3 inflammasome activation and subsequent increased IL-1β production in BV2 cells. Furthermore, we show that endocytosis of α-Syn by BV2 cells is required for the activation of NLRP3 inflammasome. Once α-Syn is endocytosed, α-Syn-containing lysosomes adopt a swollen morphology and underwent structural damage, which in turn increases the expression of lysosomal protease cathepsin B in the cytoplasm. This process seems to be causally involved in the initiation of the α-Syn-induced cascade activation of NLRP3/caspase-1/IL-1β axis since we find that IL-1β release depends on the lysosomal cathepsin B. Our results indicate that cathepsin B acts as an ‘upstream’ of NLRP3 rather than influencing the production of pro-IL-1β or mature IL-1β. Therefore, cathepsin B serves as an intermediary among α-Syn endocytosis, lysosomal damage and microglial IL-1β generation.

In addition to lysosome destabilization, cellular stress, including potassium efflux and generation of ROS, has been demonstrated to serves as a ‘bridging’ mechanism of microbial proteins induced activation of NLRP3 inflammasome [29]. For example, endocytosis of exogenous activators such as silica and asbestos by pulmonary macrophages results in NLRP3 inflammasome activation involving ROS and lysosome destabilization, in turn leading to silicosis and asbestosis, respectively [30]. Furthermore, it has been known that NLRP3, ASC and caspase-1 are preferentially expressed in adipose-tissue-infiltrating macrophages where the saturated fatty acid palmitate and lipotoxic ceramides trigger NLRP3 inflammasome activation through a mechanism that involves defective autophagy and the accumulation of mitochondrial ROS [31]. In agreement with previous findings, we show that endocytosis of α-Syn by BV2 cells induces a dramatic suppression of autophagy and a raise of ROS production. Given that AMPK is a crucial regulator in controlling autophagy, and α-Syn exhibits inhibition in AMPK phosphorylation, we further treated BV2 cells with AMPK activator AICAR to explore the effect of α-Syn on autophagy and ROS generation. Notably, we find that AMPK is responsible for the impairment of α-Syn in autophagy function and ROS production. Therefore, we conclude that α-Syn triggers mitochondrial ROS production and subsequently activates NLRP3 inflammasome via inhibiting AMPK-mediated autophagy in BV2 cells.

α-Syn accumulates as fibrillar aggregates in pathologic hallmark features in affected brain regions of PD patients, most notably in nigral dopaminergic neurons [32]. It has been demonstrated that microRNA-7 (miR-7), which is expressed mainly in neurons, represses α-Syn protein levels through the 3′-untranslated region (UTR) of α-synuclein mRNA. Importantly, miR-7-induced down-regulation of α-Syn protects DA neurons against oxidative stress [20]. These findings indicate that α-Syn production is finely controlled by miR-7. In the present study, we show a decreased miR-7 expression in the midbrain of MPTP-induced PD model mice, which possibly contributes to increased α-Syn accumulation. Interestingly, we find that miR-7 is also expressed in both primary cultured microglia and BV2 cells. We thus presume that miR-7 may directly regulate NLRP3 expression in microglia while regulates α-Syn in neurons. This hypothesis is supported by bioinformation prediction software. Therefore, we further performed dual luciferase reporter assays, and our result confirms that *Nlrp3* is a target gene of miR-7. Moreover, miR-7 mimics effectively inhibited LPS plus ATP/MSU-induced increase in NLRP3 expression and inflammasome activation while anti-miR-7 exacerbated the effect of LPS plus ATP/MSU on NLRP3 inflammasome. Most importantly, stereotactic injection of miR-7 mimics markedly inhibited NLRP3 inflammasome-mediated neuroinflammation in mouse brain and protected DA neurons against degeneration. Taken together, our findings indicate that reduced miR-7 either directly leads to NLRP3 upregulation or indirectly activates NLRP3 inflammasome via α-Syn stimulus during the process of PD. Certainly, some other miRs may be changed in MPTP model and these miRs may also target to *Nlrp3* mRNA. miR-7 may share the role in regulating NLRP3 expression with other miRNAs in PD pathogenesis.

## Conclusions

We demonstrate that NLRP3 inflammasome activation is closely associated with PD pathogenesis. As an endogenous danger signal, α-Syn activates NLRP3 inflammasomes through microglial endocytosis and subsequent lysosomal cathepsin B release and ROS accumulation. Notably, we unravel that miR-7 targets *Nlrp3* expression besides α-Syn and modulates NLRP3 inflammasome activation. Most importantly, stereotactical injection of miR-7 mimics into mouse striatum attenuated DA neuronal degeneration accompanied by the amelioration of microglial activation in MPTP-induced PD model mice. Our findings provide a direct link between NLRP3 inflammasome activation and PD pathogenesis, which will give us an insight into the potential of miR-7 and NLRP3 inflammasome in terms of opening up novel therapeutic avenues for neurodegenerative diseases including PD.

## Methods

The study protocol was approved by the Institutional Animal Care and Use Committee of Nanjing Medical University. All clinical patients gave written, informed consent to participate before screening. The study was ethically approved by the Medical Ethics Committee of Nanjing Medical University Hospital.

### Animals and reagents

α-Syn-A53T over-expressed [tg(Prnp-SNCA*A53T)83Vle/J] mice were purchased from Model Animal Resource Center (MARC, Nanjing, China). Caspase-1 knockout (Casp1tm1Sesh/LtJ) mice were purchased from MMRRC (USA). All animals were kept in cages with constant temperature (25 °C) and humidity and were exposed to a 12:12-h light–dark cycle with unrestricted access to tap water and food.

MPTP, probenecid, ATP, monosodium urate (MSU), lipopolysaccharide (LPS), cytochalasin D, AICAR, α-Syn A53T human (S1071) and α-Syn human (S7820) were purchased from Sigma-Aldrich (St. Louis, MO, USA). LysoTracker red and Hoechst 33258 were purchased from Invitrogen. A Specific inhibitor of cathepsin B (Z-FA-FMK) was purchased from Santa Cruz. The caspase-1-specific inhibitor z-YVAD-fmk (benzyloxycarbonyl–Tyr-Val-Ala-Asn–fluoromethylketone) was purchased from BioVision Research Products.

### Animal model and drug administration

Each mouse genotype (wild type and A53T^tg/tg^, male, aged 4-5 months) was divided into control and MPTP/p groups. The chronic MPTP intoxication protocol was similar to that described previously [33]: 20 mg/kg MPTP dissolved in saline was injected subcutaneously followed by 250 mg/kg DMSO-dissolved probenecid injection intraperitoneally at 1 h interval every 3.5 days over a period of 5 weeks. Control mice were treated with saline and probenecid. Animals were sacrificed one week after the final injection.

### Immunohistochemical studies and quantitative evaluation

Immunostaining method was described in the previous publication [34]. Images were observed and photos were taken under a confocal microscope (Axiovert LSM510, Carl Zeiss Co., Germany). For cell quantification in in-vivo studies, the number of TH and Iba-1-immunoreactive (Iba-1-ir) cells in the SNc of the midbrain was assessed using the optical fractionator (Stereo Investigator 7, MBF bioscience, Williston, VT, USA). Briefly, the regions of SNc in the midbrain sections were outlined at low magnification (40×). For TH-immunoreactive (TH-ir) neurons, the counting frame size was 40 μm × 40 μm and the sampling grid size was 80 μm × 80 μm. For Iba-1-ir microglias, the counting frame size was 50 μm × 50 μm and the sampling grid size was 100 μm × 100 μm. All stereological analyses were performed under the 200× objective of an Olympus BX52 microscope (Olympus America Inc., Melville, NY, USA). Within one counting frame, positive cells counted must show both TH/Iba-1 staining in the cell body and blue staining in the nuclei, and the nuclei does not touch or cross the red avoidance lines of the counting frame. The sampling scheme was designed to have coefficient of error (CE) less than 10 % in order to get reliable results. The total number of TH-ir neurons and Iba-1-ir microglia in entire extent of SNpc were counted from four mouse brains per group. Each brain contain 12 serial sections at a three intervals. The stereologer was blinded to all genotype and treatment groups for each experiment.

### Immunofluorescence

For NeuN and IBA-1/α-Syn co-labeled immunofluorescence, midbrain sections were treated for DNA denaturation but without H_2_O_2_ disposition. At the end of incubation in PBS/0.1 % Triton X-100/5 % BSA for 1 h, the sections were incubated with polyclonal anti-NeuN (1:250, mouse, Chemicon), anti-Iba-1 (1:1000, rabbit, Santa cruz) and anti-α-Syn protein (1:800, B&D) at 4 °C overnight followed by secondary antibody for 1 h at room temperature (22 ± 2 °C). Fluorescent mounting media was applied before placing coverslips and wet-mounted sections were dried in the dark. For visualization and photography, specimens were observed under Microbrightfield Stereo Investigator software (Williston, VT, USA).

### Western blotting analysis

The methods were described in our previous publication [34]. Different primary antibodies (rabbit anti-AMPK, LC3, p65, H3 and goat anti-cathepsin B, Cell Signaling Technology; goat anti-NLRP3 and rabbit anti-caspase-1, Santa Cruz; goat anti-IL-1 β, R&D Systems) and corresponding secondary antibody (1:1000) were used in the present study.

### Inflammatory factors analysis

The amounts of IL-1β in the serum of PD patients, midbrain of PD model mice and supernatant of BV2 cells were determined with a human or mouse IL-1β enzyme-linked immunosorbent assay (ELISA) kit (BioSource International, USA). Caspase-1 levels in patient serum were detected by ELISA kit (Excell, Shanghai, China). Briefly, the blood samples of twelve PD patients (age 63 ~ 78-year old, evenly divided between men and women) without drug therapy and twelve healthy controls were collected in the present study. Blood samples were kept at room temperature for 4 h and centrifuged at 3000 g for 10 min before storing. Serum was then aliquoted and stored at -80 °C until further evaluation. Caspase-1 and IL-1β levels in the serum were measured by ELISA with monoclonal antibody and half-dilution was performed according to kit instructions. The sensitivity of the ELISA was 5 pg/ml for human caspase-1 and IL-1β.

### Cell culture and treatment

Mesencephalic neuronal and microglial primary cultures were performed according to our previously described protocol [34]. Murine BV-2 microglial cells were maintained in modified Dulbucco’s eagle medium (DMEM) supplemented with 10 % fetal bovine serum and antibiotics at 37 °C in a humidified incubator under 5 % CO_2_. Cells were treated with monomer of WT α-Syn human or A53T α-Syn human for 24 h. Primary mesencephalic neurons were treated with condition medium (CM) for 48 h generated from caspase^+/+^ or caspase-1^-/-^ microglia in the absence or presence of WT/A53T-a-Syn (10 μg/ml, 24 h).

### Intracellular ROS measurement

Cellular ROS production was assessed using the ROS-specific probe 2′7′-dichlorodihydrofluorescin diacetate (H2DCFDA, BioChemica, Fluka). BV-2 cells were treated with cytochalasin D or AICAR for 1 h before stimulation with α-Syn A53T human or α-Syn human. After 24 h, cells were loaded for 30 min at 37 °C with 10 μM H2DCFDA and washed twice with PBS. Cellular fluorescence was monitored on a Fluoroskan Ascent fluorometer (Labsystems, Helsinki, Finland) using an excitation of 485 nm and emission of 538 nm.

### Immunocytochemistry

BV2 were treated with cytochalasin D for 1 h before stimulation with α-Syn A53T human or α-Syn human for 24 h. Cells were fixed with PFA 4 % in PBS for 15 min and then washed three times with PBS. After permeabilization with Triton X-100 and blocking with 1 % BSA in PBS, cells were incubated with primary antibodies (α-synuclein, 1:1000) overnight at 4 °C. After washing with PBS, cells were incubated with secondary antibodies (Invitrogen) in 1 % BSA-PBS for 1 h and washed three times. At last cells were incubated with Hoetchst 33258 for 5 min and rinsed in PBS. Cells were then imaged by confocal microscopy (Zeiss LSM510).

### Living cell image system

For evaluation of lysosomal damage, cells were incubated for 30 min with Lyso Tracker red (dilution, 1:10000) and then were stimulated with α-Syn A53T human or α-Syn human. Alexa Fluor 488–conjugated goat anti-mouse, Alexa Fluor 568–conjugated donkey anti-goat, Alexa Fluor 647–conjugated goat anti-rat and Alexa Fluor 488–conjugated goat anti-rat were purchased from Invitrogen. Lysosomal damage was assessed by Living cell system (Perkin Elmer, USA).

### Oligonucleotide Transfection

MiR-7 (sense: 5′-UGGAAGACUAGUGAUUUUGUUGU-3′, antisense: 5′-AACAAAAUCACUAGUCUUCCAUU-3′), control miR (sense: 5′-UUCUCCGAACGUGUCACGUTT-3′, antisense: 5′-ACGUGACACGUUCGGAGAA TT-3′), anti-miR-7 (5′-ACAACAAAA UCACUAGUCUUCCA-3′) and anti-miR control (5′-CAGUACUUUUGUGUAGUACA A-3′) were purchased from GenePharm (Shanghai, China). miR-7, anti-miR-7 and corresponding control miRNA were complexed respectively with Lipofectamin 2000 (Invitrogen) according to manufacturer’s instructions. Transfection efficiency was evaluated by qRT-PCR. For experiments, BV2 cells were transfected with miR-7, anti-miR-7 and corresponding control miRNA for 6 h, followed by stimulation with LPS (100 ng/ml) and then pulsed with 5 mM ATP for 30 min or 250 mg/ml monosodium urate (MSU) for 6 h.

### qRT-PCR analysis of microRNA expression

Total RNA was prepared using TRIZOL reagent (Invitrogen Life technologies, USA). For analysis of miRNA expression, stem-loop primer SYBR Green quantitative real time-PCR was performed. The relative expression was calculated using the ΔC_T_ method as described elsewhere [35] and normalized to uniformly expressed U6. The stem-loop primer sequence for reverse transcription was as follows: 5′-AGCATTCGTCTCGACACAGCAACAAAATC-3′ and the generated cDNA was amplified with these primers (5′-TGACTCTGCTGGAAGACTAGTGAT-3′ and 5′-TAGAGCATTCGTCTCGACACAG-3′). All qRT-PCRs were performed in triplicates, and the data are presented as mean ± standard error (S.E.M).

### Dual Luciferase Reporter Assays

Luciferase reporter assays were performed using the psiCHECK2-3′UTR vector. Cells were grown to 70 % confluence in 24-well plates and co-transfected with psiCHECK2-3′UTR plus miR-7 mimics or negative control mimics as described above and previously [36]. Cells were incubated with the transfection complex for 24 h followed by luciferase reporter assay using the Dual Luciferase Assay System (Promega). Renilla luciferase activity was normalized to firefly luciferase activity. Cell lysates were subjected to luciferase activity measurement according to the manufacturer’s instructions.

### Treatment of PD model mice with miR-7 mimics

Briefly, anesthetized mice were positioned in a stereotaxic apparatus, and 2 μl of phosphate-buffered saline containing 0.5 nmol of miR-7 mimics (sense: 5′-UGGAAGACUAGUGAUUUUGUUGU-3′, antisense: 5′-AACAAAAUCACUAGUC UUCCAUU-3′) or a scrambled sequence control miR (sense: 5′-UUCUCCGAACGUGUCACGUTT-3′, antisense: 5′-ACGUGACACGUUCGGAGAATT-3′; GenePharm, Shanghai, China) were injected over 8 min into the striatum (AP: +0 mm, ML: -2.0 mm, DV: -4.0 mm). Control mice received an equal volume of vehicle.

### Statistical analysis

All data were presented as mean ± SEM. Statistical significance was assessed with one–way analysis of variance followed by the *post hoc* Student-Newman-Keuls test between control and samples treated with various factors. Differences with P-values less than 0.05 were considered statistically significant.

## Additional files

Additional file 1: Figure S1. α-Syn activates NF-κB and NLRP3 inflammasome in BV2 cells. A53T mutant and wide-type α-Syn induce activation of NF-κB in a concentration-dependent manner (a) and statistical analysis reveals the changes of p65 (b) and proIL-1β (c) protein levels. Data are presented as the mean ± S.E.M from four independent experiments. Statistical analysis of Western blotting shows that α-Syn upregulates the expressions of NLRP3 (d) and caspase-1 (f) but has no effect on pro-caspase-1 production (e). Data are presented as the mean ± S.E.M from four independent experiments. * *p* < 0.05, ** *p* < 0.01 vs. control group. (TIF 2210 kb) Additional file 2: Figure S2. Aggregated α-syn activates NLRP3 inflammasome in BV2 cells. (a) Aggregated form of WT- and A53T α-Syn is identified by Western blotting. (b) Representative blots for the effects of aggregated WT- and A53T α-Syn on inflammasome activation and statistical analysis reveals the changes of NLRP3 (c) and caspase-1 (d) protein levels. (e) ELISA shows the release of IL-1β into supernatants of BV2 cells induced by aggregated WT- and A53T α-Syn. Data are presented as the mean ± S.E.M from four independent experiments. * *p* < 0.05, ** *p* < 0.01 vs. control group. (TIF 3525 kb) Additional file 3: Figure S3. α-Synuclein activates NLRP3 inflammasome through autophagy impairment and ROS accumulation in BV2 cells. Both WT and A53T α-Syn inhibit AMPK phosphorylation (a) and the ratio of LC3-II to LC3-I (b) in BV2 cells. AICAR, a specific activator of AMPK, attenuates the inhibition of α-Syn on AMPK and autophagy. Data are presented as the mean ± S.E.M from four independent experiments. Furthermore, either cytochalasin D or AICAR inhibits WT or A53T α-Syn stimulated intracellular ROS accumulation (c-d). Data are presented as the mean ± S.E.M from four independent experiments. AICAR abolishes the increase of cathepsin B induced by WT- or A53T-α-synuclein (e). Consequently, AICAR restores the increase of caspase-1 mature and subsequent IL-1β release induced by α-Syn (f-g). Data are presented as the mean ± S.E.M from four independent experiments. * *p* < 0.05, ** *p* < 0.01 vs. control group, # *p* < 0.05, ## *p* < 0.01 vs. α-Syn treatment group. (TIF 5794 kb) Additional file 4: Figure S4. The effects of miR-7 and anti-miR-7 on NLRP3 inflammasome activation in BV2 cells. (a) Upregulated α-Syn expression is detected in the midbrain of MPTP/p mice. Data are presented as the mean ± S.E.M from four independent experiments. (b) Transfection efficiency of miR-7 in BV2 cells. Data are presented as the mean ± S.E.M from three independent experiments. (c) Transfection of miR-7 into BV2 cells fails to affect caspase-1 expressions without other stimulus. Data are presented as the mean ± S.E.M from three independent experiments. (d) Transfection efficiency of anti-miR-7 in BV2 cells. Data are presented as the mean ± S.E.M from three independent experiments. (e) Anti-miR-7 has no effect on caspase-1 maturation. Data are presented as the mean ± S.E.M from three independent experiments. (f) Neither miR-7 mimics nor miR-7 inhibitor impacts IL-1β production and release in BV2 cells. Data are presented as the mean ± S.E.M from four independent experiments. * *p* < 0.05, ** *p* < 0.01 vs. NC group, # *p* < 0.05 vs. corresponding NC plus ATP or MSU group. (TIF 1683 kb)

## Abbreviations

AICAR: 5-amino-1-β-D-ribofuranosyl-imidazole-4-carboxamide
CM: condition medium
CNS: central nervous system
DA: dopaminergic
DAMPs: danger-associated molecular patterns
DMEM: modified Dulbucco’s eagle medium
IL-1β: interleukin-1β
LPS: lipopolysaccharide
miR: microRNA
MPTP: 1,2,3,6-methyl-phenyl-tetrahydropyridine/probenecid
MSU: monosodium urate crystals
NLRP3: nod-like receptor protein 3
PAMPs: pathogen-associated molecular patterns
PD: Parkinson’s disease
R-Luc: Renilla luciferase
SNc: substantia nigra compacta
TH: Tyrosine hydroxylase
TLR: Toll-like receptor
UTR: untranslated region.
α-Syn: α-Synuclein
