# Supplementary figures and images for: MicroRNA-7 targets Nod-like receptor protein 3 inflammasome to modulate neuroinflammation in the pathogenesis of Parkinson’s disease
Source: Mol Neurodegener. 2016 Apr 16;11:28. doi: 10.1186/s13024-016-0094-3 (PMC4833896; doi:10.1186/s13024-016-0094-3)

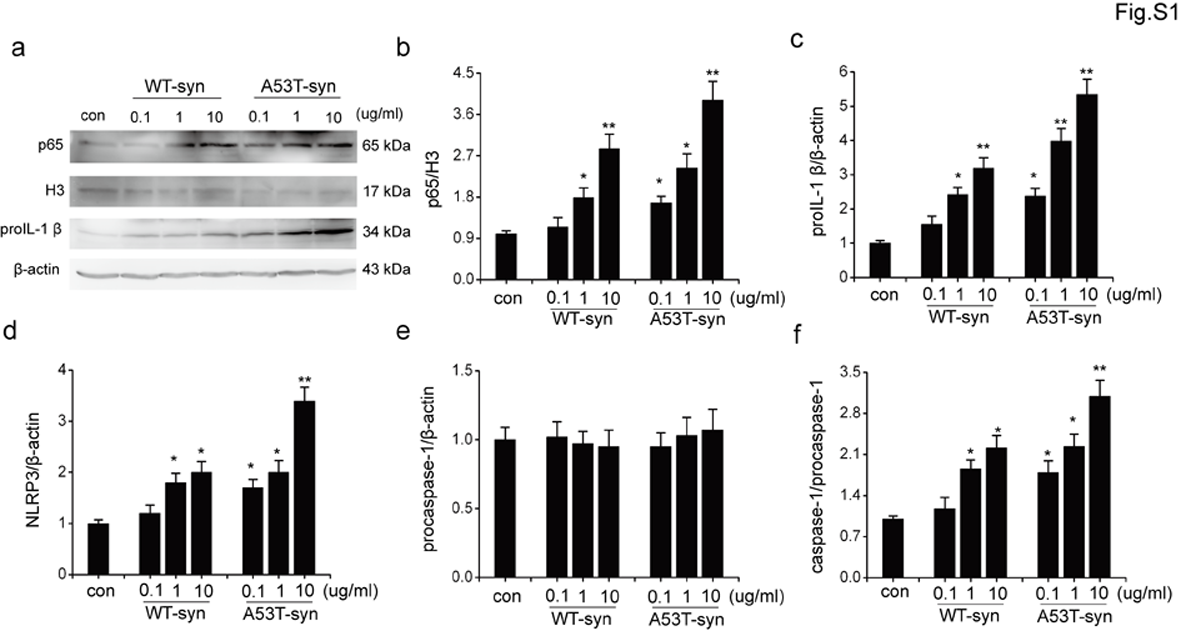

Supplement: Additional file 1: Figure S1. — α-Syn activates NF-κB and NLRP3 inflammasome in BV2 cells. A53T mutant and wide-type α-Syn induce activation of NF-κB in a concentration-dependent manner (a) and statistical analysis reveals the changes of p65 (b) and proIL-1β (c) protein levels. Data are presented as the mean ± S.E.M from four independent experiments. Statistical analysis of Western blotting shows that α-Syn upregulates the expressions of NLRP3 (d) and caspase-1 (f) but has no effect on pro-caspase-1 production (e). Data are presented as the mean ± S.E.M from four independent experiments. * p < 0.05, ** p < 0.01 vs. control group. (TIF 2210 kb) [file 13024_2016_94_MOESM1_ESM.tif]

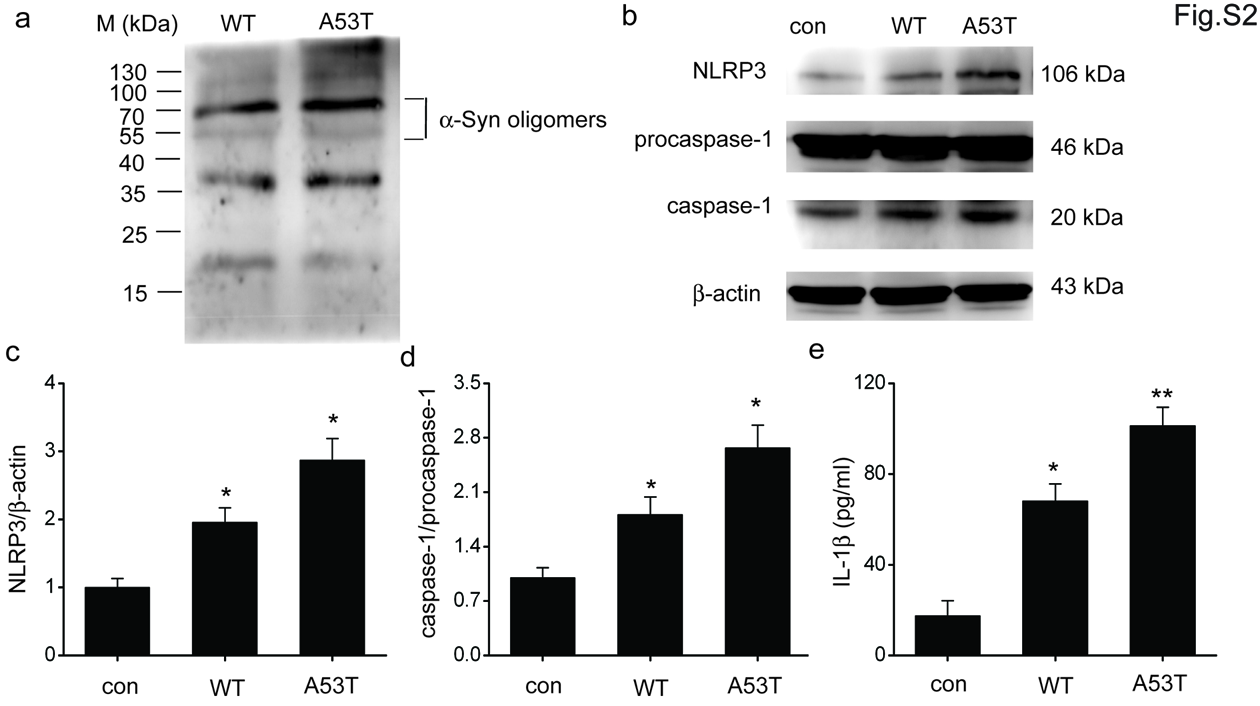

Supplement: Additional file 2: Figure S2. — Aggregated α-syn activates NLRP3 inflammasome in BV2 cells. (a) Aggregated form of WT- and A53T α-Syn is identified by Western blotting. (b) Representative blots for the effects of aggregated WT- and A53T α-Syn on inflammasome activation and statistical analysis reveals the changes of NLRP3 (c) and caspase-1 (d) protein levels. (e) ELISA shows the release of IL-1β into supernatants of BV2 cells induced by aggregated WT- and A53T α-Syn. Data are presented as the mean ± S.E.M from four independent experiments. * p < 0.05, ** p < 0.01 vs. control group. (TIF 3525 kb) [file 13024_2016_94_MOESM2_ESM.tif]

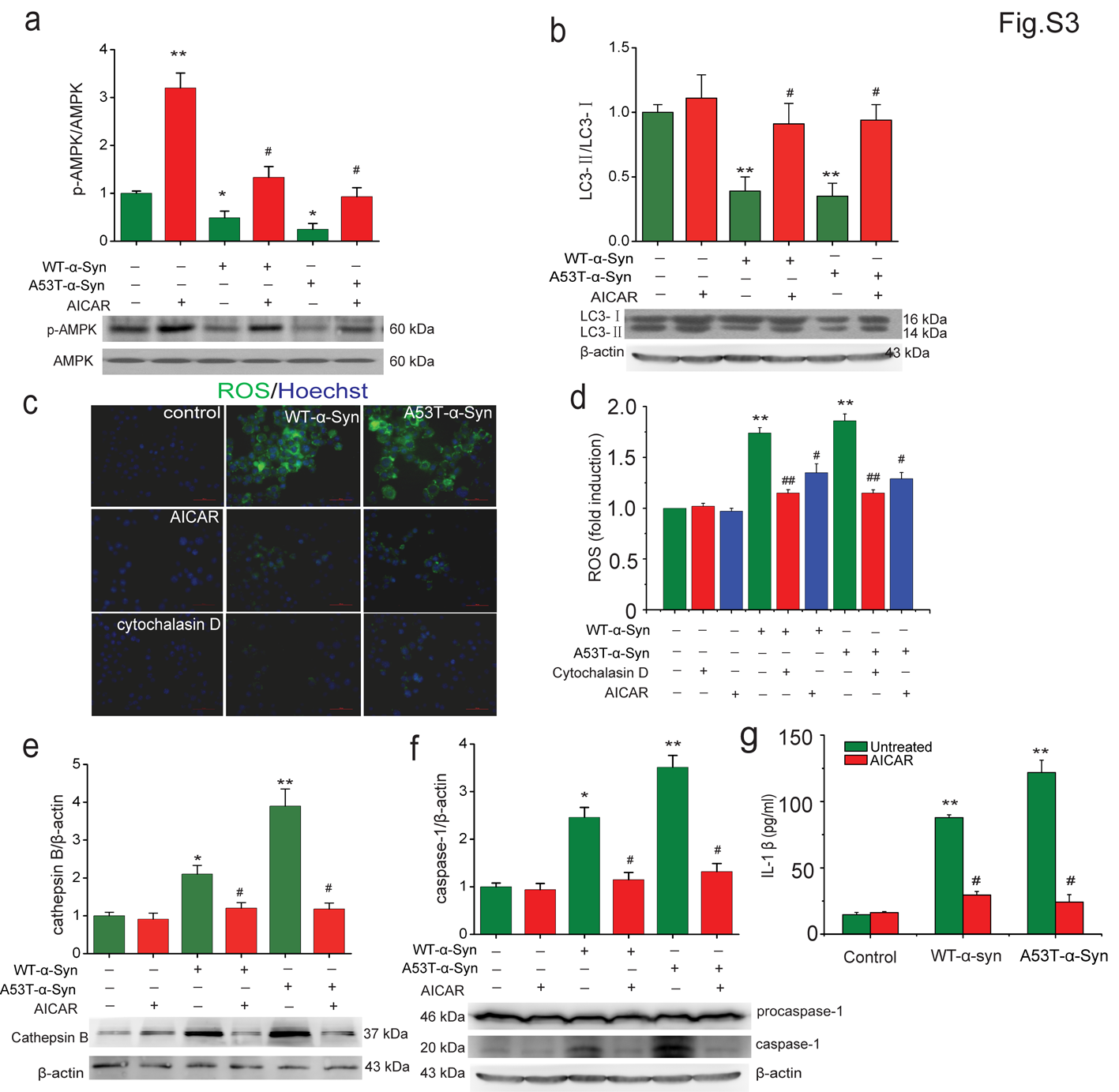

Supplement: Additional file 3: Figure S3. — α-Synuclein activates NLRP3 inflammasome through autophagy impairment and ROS accumulation in BV2 cells. Both WT and A53T α-Syn inhibit AMPK phosphorylation (a) and the ratio of LC3-II to LC3-I (b) in BV2 cells. AICAR, a specific activator of AMPK, attenuates the inhibition of α-Syn on AMPK and autophagy. Data are presented as the mean ± S.E.M from four independent experiments. Furthermore, either cytochalasin D or AICAR inhibits WT or A53T α-Syn stimulated intracellular ROS accumulation (c-d). Data are presented as the mean ± S.E.M from four independent experiments. AICAR abolishes the increase of cathepsin B induced by WT- or A53T-α-synuclein (e). Consequently, AICAR restores the increase of caspase-1 mature and subsequent IL-1β release induced by α-Syn (f-g). Data are presented as the mean ± S.E.M from four independent experiments. * p < 0.05, ** p < 0.01 vs. control group, # p < 0.05, ## p < 0.01 vs. α-Syn treatment group. (TIF 5794 kb) [file 13024_2016_94_MOESM3_ESM.tif]

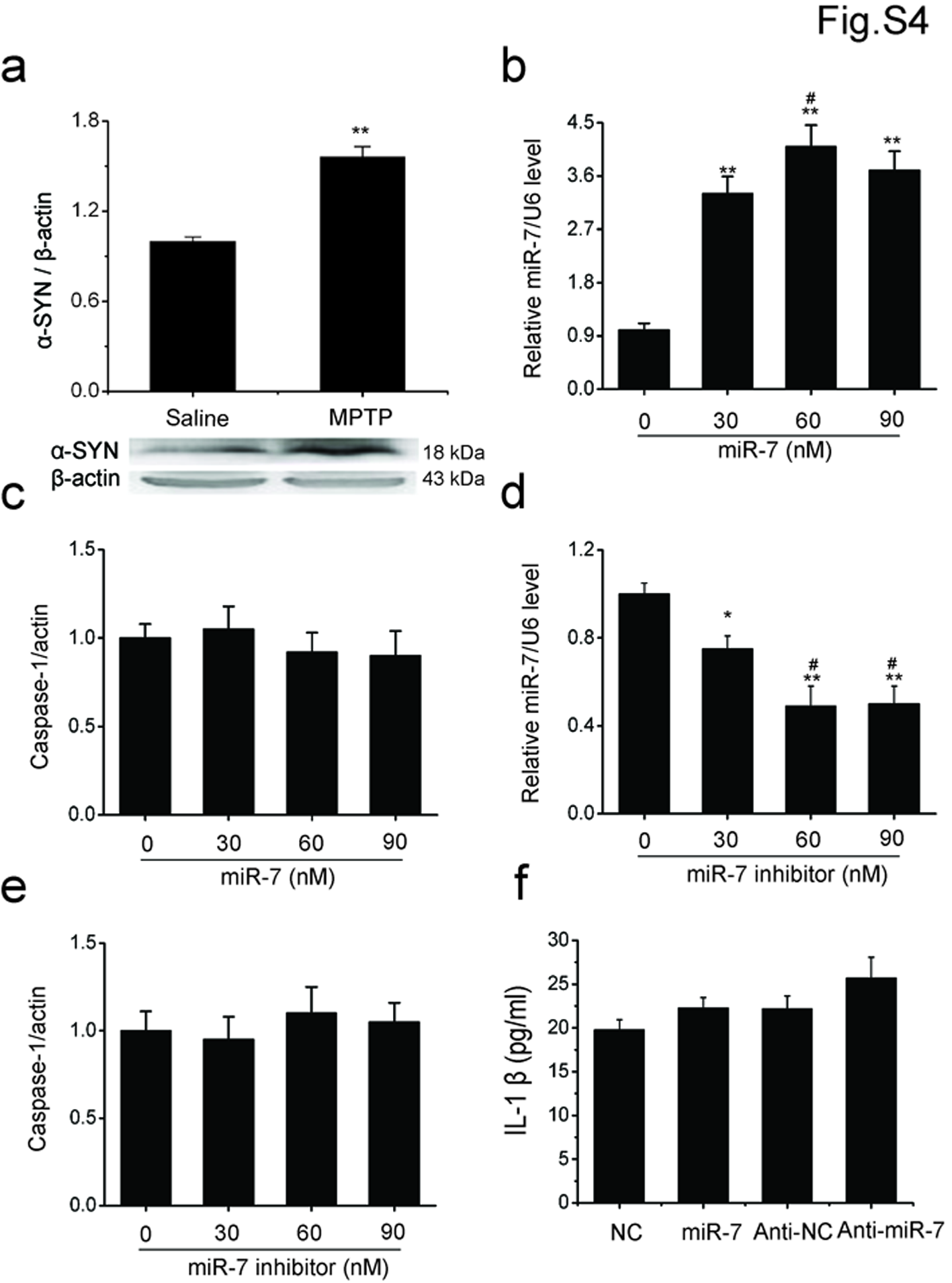

Supplement: Additional file 4: Figure S4. — The effects of miR-7 and anti-miR-7 on NLRP3 inflammasome activation in BV2 cells. (a) Upregulated α-Syn expression is detected in the midbrain of MPTP/p mice. Data are presented as the mean ± S.E.M from four independent experiments. (b) Transfection efficiency of miR-7 in BV2 cells. Data are presented as the mean ± S.E.M from three independent experiments. (c) Transfection of miR-7 into BV2 cells fails to affect caspase-1 expressions without other stimulus. Data are presented as the mean ± S.E.M from three independent experiments. (d) Transfection efficiency of anti-miR-7 in BV2 cells. Data are presented as the mean ± S.E.M from three independent experiments. (e) Anti-miR-7 has no effect on caspase-1 maturation. Data are presented as the mean ± S.E.M from three independent experiments. (f) Neither miR-7 mimics nor miR-7 inhibitor impacts IL-1β production and release in BV2 cells. Data are presented as the mean ± S.E.M from four independent experiments. * p < 0.05, ** p < 0.01 vs. NC group, # p < 0.05 vs. corresponding NC plus ATP or MSU group. (TIF 1683 kb) [file 13024_2016_94_MOESM4_ESM.tif]
